# Hierarchy Depth in Directed Networks

**DOI:** 10.3390/e24020252

**Published:** 2022-02-08

**Authors:** Krzysztof Suchecki, Janusz A. Hołyst

**Affiliations:** 1 Center of Excellence for Complex Systems Research, Faculty of Physics, Warsaw University of Technology, Koszykowa 75, 00-662 Warszawa, Poland; 2 ITMO University, Kronverkskiy Prospekt 49, 197101 St. Petersburg, Russia

**Keywords:** hierarchy, directed graph, complex network

## Abstract

In this study, we explore the depth measures for flow hierarchy in directed networks. Two simple measures are defined—rooted depth and relative depth—and their properties are discussed. The method of loop collapse is introduced, allowing investigation of networks containing directed cycles. The behavior of the two depth measures is investigated in Erdös-Rényi random graphs, directed Barabási-Albert networks, and in Gnutella p2p share network. A clear distinction in the behavior between non-hierarchical and hierarchical networks is found, with random graphs featuring unimodal distribution of depths dependent on arc density, while for hierarchical systems the distributions are similar for different network densities. Relative depth shows the same behavior as existing trophic level measure for tree-like networks, but is only statistically correlated for more complex topologies, including acyclic directed graphs.

## 1. Introduction

The concept of hierarchy has been used for systems described with networks for a significant time and for different purposes. It has been considered in the context of elections [1], investigation of road network geometry [2], and neural networks [3]. More recently, the concept of hierarchy has been used for social systems [4,5,6,7,8], artificial neural networks [9], financial markets [10], communication networks [11], and the structure of cities [12]. It has also been used on a more theoretical level to distinguish and explain specific network properties [13]. While the concept of hierarchy has been extensively used, it is continuously lacking a definition. There are papers that attempt to remedy that by introducing more formal definitions and measures [14,15,16]. The paper by Corominas-Murtra [16] introduces a definition of causal graphs and perfect hierarchical structure, and introduces a measure of hierarchy that is based on the similarity of a given graph to a perfect hierarchy. The paper by Mones et al. [15] distinguishes between three types of hierarchy: order (as in [4,5,6,7,8]), nested (as in [13]), and flow (as in [15,16,17]).

The order hierarchy is a simple ordering of elements of a set along a one-dimensional axis. The nested hierarchy is tied to the community structure and multi-scale organization of the network. The flow hierarchy is basically a causal structure, with (using the tree analogy) the “root” being the origin of all signals or flows, and “leaves” being simply receivers. In this paper, we focus on the flow hierarchy. As pointed out in [15], order hierarchy can be seen as simple flow hierarchy, and nested hierarchy can be represented by a flow hierarchy. In our work, we intend not to make another definition of hierarchy, but to measure a single aspect of it: its depth. The hierarchical relations defining or that are present in the network may affect opinion and information spreading [18,19], learning process [20], and also involuntary spread of pathogens [21]. The issue of the overall network direction is also important for food webs and supply networks [17].

In the case of perfectly hierarchical networks [16] it is trivial to describe the depth of its nodes—it is simply the distance from the top node to a given node. The situation is less clear if the topology of the network is more complex. Even if there are no loops in the network, its depth may still not be obvious, with basal nodes positioned at different distances from the top, the root node, or existence of multiple top-level nodes. Adding multiple parallel paths makes the problem even more complicated and depending on definition of the depth may lead to frustration, as paths of different lengths between two nodes may mean a necessity to use two different depth differences at the same time. Finally, the presence of directed loops means it is impossible for a depth measure that is in agreement with all links to exist. Recently there has been substantial work in assigning a hierarchy to directed graphs with strongly connected components. These methods usually rely on a minimalization of a measure, called agony [22], trophic incoherence [17], or hierarchical incoherence [23]. They assign levels that are by necessity in violation of the directed network structure and often require substantial computational effort to calculate if the networks are large. In addition, their results may be hard to interpret outside of designating which nodes are higher and which are lower in a hierarchy. The aim of our work is not to disentangle the strongly connected component and assign levels to the nodes it consists of, but to describe the structure that is already present in the network. We would also like our measures to have a clear definition and interpretation that goes beyond the relation of which node is higher and which node is lower.

We introduce two simple depth measures for directed networks and discuss their behavior in synthetic and some real network examples as well as the impact of directed cycles on those. The rooted depth of a given vertex is the length of a shortest path from a specified root to the given vertex, and is a simple extension of hierarchy levels found in perfect hierarchies. Depending on a chosen root, this depth varies, and for some vertices it may be undefined, if a path from the root does not exist. The relative depth of a given vertex is defined by the arcs to other vertices. The depth of a target vertex of an arc is always higher than that of the source vertex. The difference of relative depth between two nodes coincides with the length of the longest directed path connecting them. Relative depth is undefined for strongly connected components, as it is impossible to assign values fully consistent with the links. To handle such graphs, we make an additional assumption that all vertices that are part of a directed cycle have the same relative depth. This follows an intuitive solution of a problem if the network represents a commended structure—no one in the cycle is in fact more important than others. By using this assumption, it is possible to describe the flow hierarchy depth in the parts of the network that are truly hierarchical and do not contain cycles.

Both depth measures have simple interpretations, with a difference related to the shortest or longest paths, and are relatively simple to determine algorithmically, which allows calculating them even in large networks.

## 2. Materials and Methods

We define depth measures for directed networks, which are composed of vertices connected by arcs (directed edges). Any undirected graph can be considered directed, with each undirected edge corresponding to two directed arcs mutually, linking both edge endpoints. The depth is defined in the context of flow hierarchies, and is therefore most meaningful for networks that have a certain overall direction of all arcs.

The rooted depth is a value defined for each vertex in a network, and is the distance of that vertex from a root vertex. This is a very intuitive definition for hierarchy and its drawback is the necessity to choose a root, which may be arbitrary for networks without a natural root.

**Figure 1:**
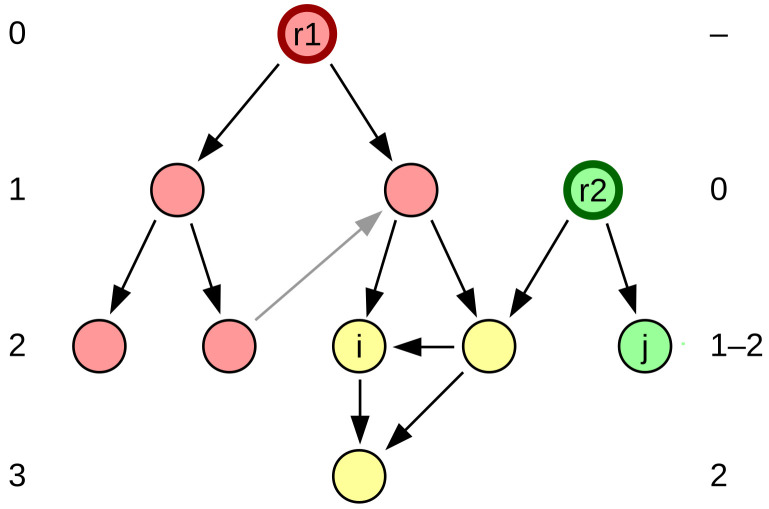
Directed graph and its rooted depth. Red vertices belong only to the out-component of root r1, while green only to the component of root r2. Yellow vertices belong to both out-components. Depth levels of vertices (left for r1, right for r2) may be different relative to each root. Vertex *i* is at depth 2 from root r1 and at depth 2 from r2—it is generally not possible to draw the depth levels consistently for rooted depth with multiple roots. Rooted depth relies on the shortest paths and ignores links not belonging to one, which are marked here in gray instead of black.

For a directed tree, the root is natural: the one vertex with outgoing arcs only. Each vertex *i* is at certain depth $d_{i}$ in the network, which we define as the distance from root to the vertex $l_{ri}$. This means that all vertices are organized into distinct depth levels. It can be easily extended for more complex networks, provided a root can be identified or defined (Figure 1), which we will call a rooted network. Since rooted depth considers distance, it effectively takes into account only the shortest paths from the root to all other vertices, ignoring the rest of them. Directed cycles in the network do not impact this measure, as any arcs that are not part of the shortest paths from the root are simply ignored.

In case there is no single natural root in the network, we may further extend the definition by considering any vertex with no incoming arcs as a root. Any graph can then be considered simply a combination of several rooted networks (Figure 1). Each root vertex has a part of the network reachable from it—an out-component of that root, where the depth is defined by distance from the root. A vertex may have several different rooted depths, one for each of the roots. In the final case, there may be no vertices with outgoing links only, in which case a root must be arbitrarily chosen, or the rooted depth cannot be calculated.

**Figure 2:**
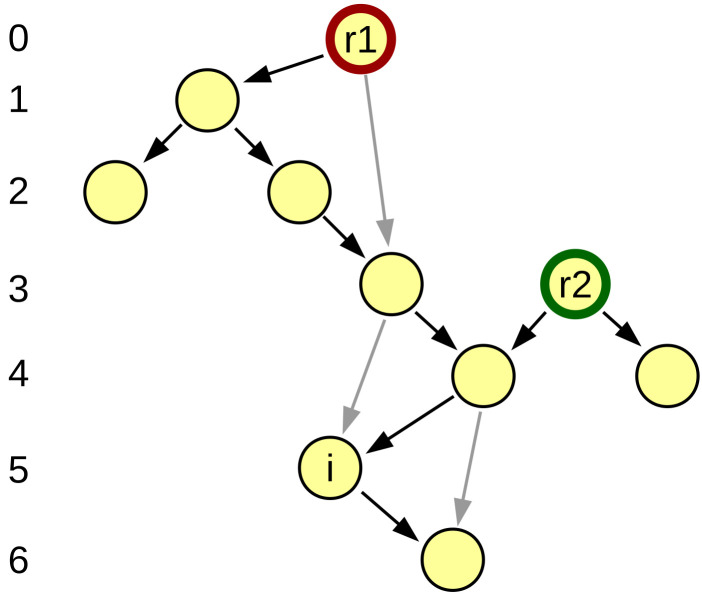
Acyclic directed graph and its relative depth. The graph is the same as in Figure 1, except the layout is adapted to relative depth values. Note that vertex *i* is at high depth 5 despite being only two arcs away from the depth 0. Two roots present in the graph, r1 and r2, are found at depths 0 and 3. All arcs always point downwards, by the definition of relative depth. Relative depth effectively only takes the longest paths into account, ignoring links that are shortcuts, marked in gray instead of black here.

The relative depth is defined as a value $d_{i}$ assigned to each vertex *i*, such that any arc ij always connects from a vertex with lower depth $d_{i}$ to a vertex with higher depth $d_{j}$, meaning that $d_{i}<d_{j}$. Effectively, the arcs act as inequalities that all must be fulfilled by the relative depth and relative depths are solutions to these inequalities. This definition is ambiguous, but it may be made unambiguous if we add further restrictions. We add the restriction that the depth difference in the arc must be as small as possible but at least 1 ($d_{j}-d_{i}\geq 1$). This leads to the relative depth difference between any two vertices *i* and *j* being equal to the length of the longest directed path ij between them, provided such path exists (Figure 2). Since the values are defined by relations only, similar to state functions like energy or entropy in physics, there is no set “zero level” and the differences between values of vertices result from the definition. For convenience, it is reasonable to set the vertex with the smallest depth as 0, obtaining similar non-negative depth levels as in the rooted depth. The relative depth definition works for directed acyclic graphs only. Defining it for a network with even a single directed cycle requires additional assumptions, similar to rooted depth without a clear root node.

**Figure 3:**
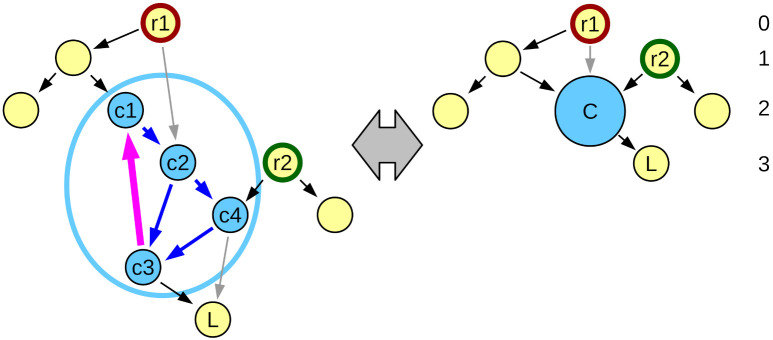
By assuming that all vertices that belong to a cycle have the same depth, it is possible to substitute them with a virtual vertex representing them, thus reducing any graph to acyclic. The figure shows the same network as in Figure 2 except for a single added arc between vertices c3 and c1 (shown in thick magenta). All vertices c1, c2, c3 and c4 (marked in blue) form two non-separated directed cycles, and thus are substituted by a single virtual vertex *C*. After calculating depth, vertices c1, c2, c3 and c4 are all assigned depth 2, as corresponding virtual vertex *C*.

To handle networks with directed cycles using relative depth, we simply define that all vertices that belong to a given cycle have the same relative depth, which supersedes the differences implied by the arcs. If we do so, we can essentially replace the whole cycle with a single vertex for purposes of calculating relative depth (Figure 3). If a vertex is a part of more than one cycle, then all vertices in both cycles will have all the same depth (transitivity of equality). In networks with many cycles, this may lead to a majority of the vertices all sharing the same depth value. We call the process of temporarily replacing all vertices in the same cycle with a single vertex a “loop collapse”. Note that it is possible to use loop collapse independently from relative depth definition, for example by using it before calculating rooted depth and potentially obtaining natural roots required by that definition where none could be found, although each of these roots may be a set of vertices sharing the same depth.

We have studied the behavior of the model on two synthetic network models: Erdös-Rényi random graphs (E-R) and directed Barabási-Albert (B-A) scale-free network, as well as the Gnutella file sharing network.

Erdös-Rényi random graph is a graph model, where each pair of vertices is connected with a fixed probability *p* that is a parameter of the model [24]. We consider the so-called quenched model, which means that the actual topology is fixed through random choices, producing a specific realization of the graph. This model is mostly uncorrelated and often serves as a null model for considering topological features in random networks. We choose the parameter $p=\langle k\rangle /\left(N-1\right)$ and consider each arc separately to obtain directed networks with a specified mean degree $\langle k_{in}\rangle =\langle k_{out}\rangle =\langle k\rangle$.

Barabási-Albert scale-free network model [25] is an evolving network model, where to create a realization one needs to grow it, starting from an initial clique of *m* vertices and add new vertices one by one until the network possesses desired *N* vertices in total. During the network growth, each new vertex creates *m* edges to existing vertices. The choice of vertex to connect to is random but preferential. The probability $P_{i}$ of connecting a new vertex to a vertex *i* equals to $P_{i}=k_{i}/\sum_{j}k_{j}$. The parameter *m* controls the network density with an average degree of $\langle k\rangle =2m$. The network is always a single connected component and features power-law degree distribution $P\left(k\right)∼k^{-3}$. We consider a directed variant with the possibility of a non-integer parameter *m* value, where each new vertex creates ⌊m⌋ or ⌈m⌉ arcs, so that the mean is equal to the specified value $\langle m\rangle$, and each vertex creates an arc from the vertex it chooses for itself. The preferential choice considers both in- and out-coming arcs to determine probability. Using this method, the arcs are always from older to newer vertices, resulting in an acyclic directed network that may possess multiple parallel paths if $\langle m\rangle >1$.

The Gnutella network is a graph representing the connections made between hosts in peer-to-peer file sharing network. Vertices represents hosts/users and arcs mean that an internet connection between the hosts have been done. The data we have used show connections made on 4 August 2002 [26].

## 3. Results

**Figure 4:**
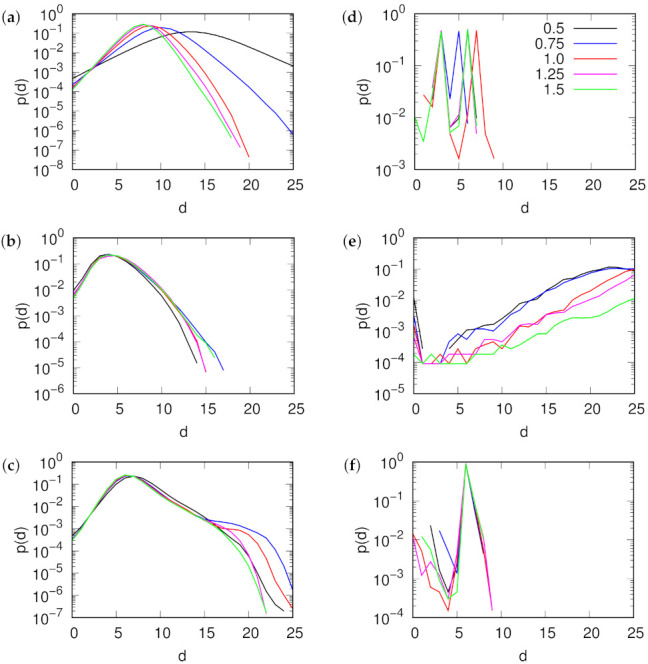
Distribution of rooted (left column **a**–**c**) and relative (right column **d**–**f**) vertex hierarchy depth in (**a**,**d**) Erdös-Rényi random graph, (**b**,**e**) directed Barabási-Albert scale-free network, (**c**,**f**) Gnutella peer-to-peer file sharing network for different network densities (mean degree $\langle k\rangle$ indicated by line color). All networks have N= 10,876 and have arcs removed at random to result in specified mean degree. Graphs for rooted depth show distributions aggregated over all possible roots (vertices with no incoming arcs), and for relative depth include loop collapse (except directed B-A, which has no directed cycles). The relative depths are shifted to match the depth of the first large peak.

We have tested some of the properties of the depth measures in synthetic and real networks—in Erdös-Rényi (E-R) random graphs, Barabási-Albert (B-A) scale-free networks, and the Gnutella file sharing network [26]. All networks are directed, have N= 10,876 vertices (number of vertices in the Gnutella network) and have part of their arcs removed at random to obtain the desired mean degree $\langle k\rangle =\langle k_{in}\rangle =\langle k_{out}\rangle$. We have calculated the rooted depth and the relative depth in the networks numerically. We produce 100 realizations of the network and calculate depth measures using algorithms based on breadth-first-search method. Obtained depth values for vertices have been aggregated over all network realizations into the distributions shown in Figure 4. In the case of Gnutella 100 the realizations mean starting from a full original network and sparsening it by random link removal with uniform distribution. For synthetic networks it means the creation of the network from scratch according to the specified model, with model parameters set to obtain a specific mean degree $\langle k\rangle$ and a size *N*. In addition, for the relative depth we have shifted all values by a constant, so that they are non-negative and the first peak coincides. This has been done for a better comparison, since the relative depth does not have a specified zero point.

The distributions of rooted depth among the vertices are shown in left column of Figure 4. While the distribution for Erdös-Rényi random graphs depends on the network density, especially in the region below the percolation threshold, it does not show significant changes for the Barabási-Albert model or for the real Gnutella network.

The distributions of relative depth show a different picture (right column of Figure 4). Both random graphs and Gnutella feature a significant amount of cycles and, as a result of loop-collapse, which is required to determine relative depth in such case, have most of the vertices at the same depth, corresponding to the strongly connected component of the graph. On the other hand, directed Barabási-Albert networks, where new vertices always had added arcs pointing towards them, show clear flow hierarchy structure. The number of vertices increases with depth, as it would in an ideally hierarchical tree. The core of the network serves as a natural root with increasingly more distant vertices being more numerous.

**Figure 5:**
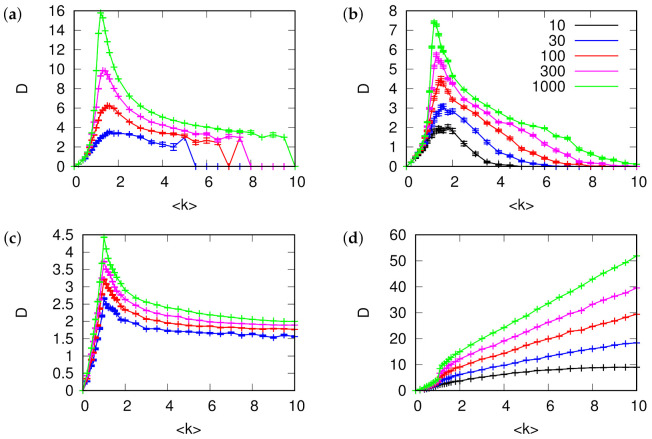
Dependence of network depth *D* (Equation (1)) on network connection density $\langle k\rangle$ for (**a**) rooted depth without loop collapse, and (**b**) relative depth with loop collapse, for the directed E-R random graph of specified size *N*, as well as (**c**) rooted depth without loop collapse, and (**d**) relative depth with loop collapse, for directed B-A networks (connections from older to newer nodes always result in the absence of directed cycles). Each point is the result of 100 network realizations and error bars (too small to be visible for most points) show the standard deviation of the calculated average value. Lines are added to guide the eye.

We also investigated the behavior of network depth *D*, which by analogy to the depth of bodies of liquid, we define as the difference between depth measure of leaf vertices ($k_{out}=0$) and root vertices ($k_{in}=0$).

(1) $$D=\frac{1}{N_{RL}}\sum_{r,l}\left(d_{l}^{\left(r\right)}-d_{r}\right)$$

where $d_{r}$ is the depth measure of a root *r*, $d_{l}^{\left(r\right)}$ is the depth measure of a leaf *l* relative (if applicable) to the root *r*, and $N_{RL}$ is the number of root-leaf pairs. We have calculated this measure similarly to the first setup. For each network size *N* and density $\langle k\rangle$ we have created a network model realization 100 times. For each realization we have calculated the global depth measure *D* (Equation (1)) and finally we have calculated the mean value and the standard deviation of the mean depth difference over all root-leaf pairs in all network realizations. The results are shown in Figure 5.

The behavior of network depth *D* in the random graphs is tied to their percolation properties. As seen in Figure 5a–c, the depth increases with network density $\langle k\rangle$ as connected clusters grow in size, and after the graph is percolated it starts to decrease as greater connectivity leads to shorter and shorter mean path lengths. Note that in case no root or leaf vertices were present, we assumed that the network depth is 0, which is responsible for the final drop of the value to zero as $\langle k\rangle$ increases. If the network is not random, but has actual flow hierarchy structure, such as the directed Barabási-Albert network, the behavior of the relative depth is significantly different (Figure 5d). Below the percolation threshold, where parallel paths are rare, it behaves almost the same. Above percolation threshold, there is a steady increase in the relative depth, as the increasing density of the network provides increasingly longer paths.

**Figure 6:**
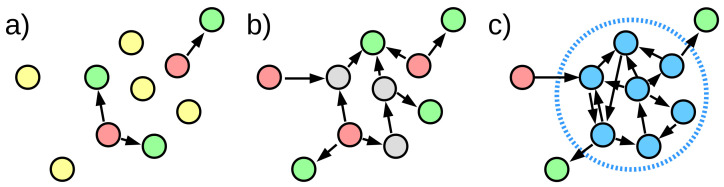
The example topology of a random network at different arc densities $\langle k\rangle$ and its relation to the depth values. (**a**) for low density $\langle k\rangle <1$, (**b**) at percolation threshold $\langle k\rangle =1$, (**c**) for high density $\langle k\rangle >1$. Roots are marked in red, leaves in green, vertices that are both at the same time in yellow, while vertices belonging to loops are blue. At (**c**), the network has a strongly connected “core” that consists of interleaving loops and therefore, under loop-collapse rules, all have the same depth. The roots and leaves are attached to that core.

The increase, peak, and plateau of the network depth *D* for Erdös-Rényi random graphs correspond to the phases of disconnected graph at low $\langle k\rangle$, percolation threshold $\langle k\rangle =1$, and higher connectivity $\langle k\rangle >1$ as shown in Figure 6.

**Figure 7:**
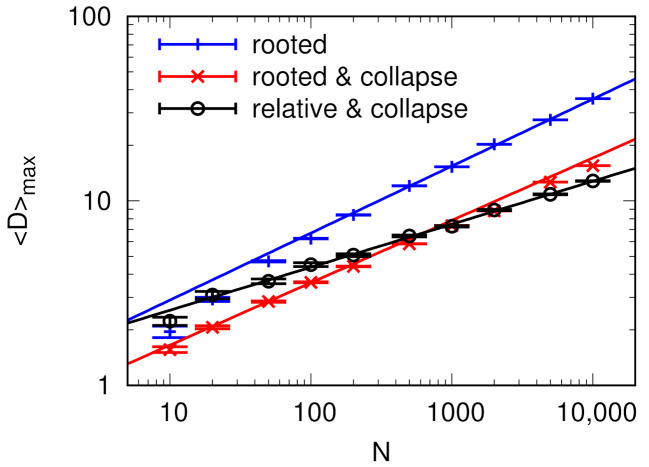
Dependence of peak network depth $D_{max}$ for E-R graphs on the graph size *N*, for rooted depth, rooted depth with loop collapse, and relative depth with loop collapse. The values were taken from the $D(\langle k\rangle )$ series with 0.1 resolution on $\langle k\rangle$, where each point is the result of 100 network realizations. The lines are fit to points N≥100 and have the slopes 0.363±0.005 (rooted), 0.328±0.008 (rooted with collapse), and 0.233±0.004 (relative with collapse).

The maximum network depth $D_{max}\left(\langle k\rangle \right)$ observed for each network size has been also plotted against network size *N*, with results shown in Figure 7. The peak heights scale sub-linearly with graph size, The relative depth peak height scales with a smaller exponent than the peak for rooted depth. Including loop-collapse does not substantially modify the behavior of the peak height for rooted depth.

**Figure 8:**
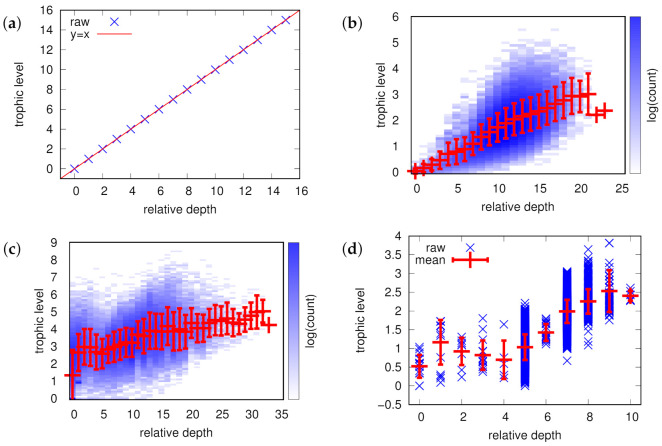
Comparison between the relative depth and the trophic levels according to the method by Mackay et al. [17] for the same nodes in (**a**) directed Barabási-Albert networks with $\langle k\rangle =1$ (a tree), (**b**) directed Barabási-Albert networks with $\langle k\rangle =1.5$, (**c**) Erdös-Rényi rangom graphs for $\langle k\rangle =1.5$, and (**d**) Gnutella network sparsened to $\langle k\rangle =1.5$. The raw data are shown in blue (points for Gnutella, histogram for others), while the red symbols show the mean value and standard deviation of trophic level distribution for nodes with a given relative depth. Data is from 100 realizations of synthetic networks and a single sparsening of the Gnutella network.

We have also compared the relative depth and trophic levels of the same vertices in different networks. Trophic levels have been calculated according to the method by MacKay et al. [17]. Similarly to previous results, we have created 100 realizations of the specific synthetic network and calculated the relative depth for all vertices, adding a constant afterwards so that values are non-negative, then calculated trophic levels for all vertices also adding a constant so that values are non-negative, and plotted the two measures against each other for each vertex. The results are shown in Figure 8. Inclusion of the constant is necessary, because both the relative depth and the trophic levels as defined by Mackay et al. have one degree of freedom for each connected network component, since the only differences between the depth/level depend on the network topology.

It is evident that for a trivial case of a tree-like network (Figure 8a) that both measures coincide exactly if one sets the same reference vertex with a depth/level equal to 0. In case the graph does not contain directed cycles, but can possess multiple parallel paths, the values are clearly correlated, but stop being equivalent (Figure 8b), especially for vertices deeper in the hierarchy. For networks with strongly connected components, the discrepancy between relative depth using loop collapse and trophic level increases, now having potentially very different values even for vertices in the top of the hierarchy (where the reference vertex with depth/level set to 0 is located) (Figure 8c). The same can be observed in real networks (Figure 8d).

## 4. Discussion

We have defined two depth measures for flow hierarchies in directed networks. The simple rooted depth is defined as the shortest path from one of the network’s roots, while the more complex relative depth is defined through the relations between vertices and the difference in depths is effectively equal to the longest path between vertices. An assumption of all vertices belonging to a directed cycle sharing the same depth allows to determine the relative depth also for networks that contain directed cycles, but it leaves strongly connected components in such topologies unresolved.

The measures provide descriptions of the hierarchical structure of the network and could be used for command structures or supply networks. Application of the measures, especially relative depth, to the supply networks could allow for a better understanding of how dependent businesses are on the rest of the network and compare the complexity of their supply chains. It may also serve as an input for methods that try to estimate impact of supply chain perturbations.

The rooted depth measures hierarchy depth locally, relative to a single specific root, while relative depth assigns depths universally and takes the whole structure of the network into account, not only the shortest paths. A clear distinction between the directed Barabási-Albert model and random graphs have been observed, especially for relative depth in loop-collapsed network, which is a narrow peak for random graphs and a wide distribution for a hierarchical directed network. Despite their differences, both measures converge to the same, intuitive values for perfect hierarchies [16]. While we have defined these for unweighted graphs, both measures can be easily extended to include a function of the arc weights (e.g., straight weight, inverse, or more complex “distance” based on arc weight). For rooted depth, the shortest paths have to include weights, as is normal for shortest paths in weighted networks. For relative depth, the minimum difference could be set to be a function of the weight instead of 1, reproducing the same behavior—the relative depth difference is equal to the weighted length of the longest path between the vertices.

It is of interest how the different measures of a similar nature compare to each other. We have compared the values of relative depth to a trophic level, a measure defined in ecology, and intended to show how high or low in food webs given species represented by a network vertex is, but also used for other purposes. In particular we have calculated trophic levels according to a method by MacKay et al. [17] with results shown in Figure 8. This method is designed to assign a hierarchy (trophic levels) to all vertices, including a strongly connected component, that minimalizes the discrepancy between trophic levels of the vertices and network topology, where a perfect difference of levels along any arc is 1 (for unweighted networks). This approach allows one to find the levels for all vertices, but by necessity some of the vertex pairs in strongly connected components have levels assigned that go against connections in the network. Both of the measures give different results and a different perspective of the system, although the correlation between these two measures can be also seen. The two measures have been developed with different aims. While methods of calculating trophic levels have been designed mostly to disentangle strongly connected components of a network and assign a hierarchy that is least in violation of the network structure, the relative depth aims to describe systems already possessing a nature of directed flow hierarchies, or at least parts of the system that do. Both measures show the same results for some simple network motifs (such as a cyclic triangle) and tree graphs (no parallel paths), but start to diverge with the addition of multiple parallel paths. The amount of calculations required to assign the relative depth is also smaller than for trophic level calculations that involve matrix algebra, especially for a larger network (10,000 vertices or more), which means they may find use in analysis of large hierarchical systems.

For random directed graphs, the global network depth—a mean of depth difference for all root-leaf pairs—behaves in a similar manner to the mean graph diameter depending on graph link density. Below the percolation threshold, it increases up to the threshold, where the trend reverses and the depth start to decrease. This relationship is supported by the peak depths $D_{max}$ close to the percolation threshold. This value scales as a power of network size (Figure 7) with exponents around 1/3, similar to the scaling of the diameter of the critically percolated largest network component $C\left(N\right)∼N^{1/3}$ [27]. The relative depth peak scales with a significantly lower exponent, but it is worth noting that since it relies on the longest, not the shortest paths, the difference is not surprising.

It is notable that the shape of the dependence of network depth on density (Figure 5) is very similar to the behavior of the Global Reaching Centrality (GRC) measure introduced by Mones et.al. [15] and is calculated for directed random graphs [28]. This is because measures react very similarly to percolation—in our case by maximizing path lengths in components and by maximizing the width of the Local Reaching Centrality (LRC) distribution and thus GRC in the other. Both rely on large acyclic components to achieve high values, explaining the correlation.

The introduced measures have several advantages and disadvantages compared to existing measures such as the trophic level [17]. The advantages include simplicity of definition, low complexity of calculations required to determine them for even a large network and an easy to understand interpretation of the measures—a difference being equal to the shortest or longest directed path, depending on whether we consider the rooted or relative depth. The main disadvantage of the measures is their reliance on an arbitrary root choice for the rooted depth and their inability to meaningfully assign relative depth values in strongly connected components. The future work related to the measures could include an analysis of depth behavior if it is extended to weighted networks and improved ways for the relative depth to cope with the directed cycles, such as disregarding the minimal amount of links required to remove all directed cycles. Note that the second future work prospect is not intended to determine depths for dense strongly connected components, as for example in [17], but to allow for studies of networks that do possess a hierarchical structure with only few contradicting links, which may be caused by noise or data errors.

## Data Availability

Publicly available datasets were analyzed in this study (Gnutella network topology). This data can be found here: https://snap.stanford.edu/data/ accessed on 12 April 2021 [26].
